# Association of subclinical hypothyroidism with metabolic syndrome components in a group of apparently healthy Syrians: a retrospective cross-sectional study

**DOI:** 10.1097/MS9.0000000000000184

**Published:** 2023-03-14

**Authors:** Zaynab Alourfi, Nermeen Hijazi, Mohammad Alsultan

**Affiliations:** Department of Internal Medicine, Faculty of Medicine, Damascus University, Damascus, Syria

**Keywords:** central obesity, euthyroid, high-density lipoprotein, metabolic syndrome, subclinical hyperthyroidism, subclinical hypothyroidism, triglycerides

## Abstract

Thyroid disorders were reported to be associated with various diseases, particularly dyslipidemia. This study aimed to assess the prevalence of thyroid disorders in a group of apparently healthy Syrians and investigate the relationship between subclinical hypothyroidism and metabolic syndrome (MetS).

**Methods:**

A retrospective, cross-sectional study was performed at Al-Assad University Hospital. Participants were healthy individuals aged 18 years and older. Data about their biochemical tests, weight, height, BMI, and blood pressure were collected and analyzed. Participants were categorized according to their thyroid tests into euthyroid, subclinical hypothyroid, subclinical hyperthyroid, and according to their BMI into normal, overweight, and obese, and according to the International Diabetes Foundation into normal and having MetS.

**Results:**

A total of 1111 participants were involved in this study. Subclinical hypothyroidism and subclinical hyperthyroidism were found in 4.4 and 1.2% of participants, respectively. The incidence of subclinical hypothyroidism was significantly increased in females and in the presence of positive antithyroid peroxidase. Subclinical hypothyroidism was significantly associated with MetS, a higher waist circumference, central obesity, and triglycerides; however, there was no correlation with high-density lipoprotein.

**Conclusion:**

The prevalence of thyroid disorders among Syrians was consistent with the results of other studies. These disorders were significantly more common in females compared to males. Add to that, subclinical hypothyroidism was significantly associated with MetS. Since MetS is a known factor for morbidity and mortality, this may raise the attention needed to perform future prospective trials to evaluate the possible benefits of subclinical hypothyroidism treatment with a low dose of thyroxin.

## Introduction

Thyroid hormones regulate metabolism and affect lipid and glucose metabolism, leading to changes in body weight and composition. Thyroid disorders (either subclinical or clinical) are reported to be one of the most common endocrine disorders, diagnosed by testing the serum thyroid-stimulating hormone (TSH) level and thyroid hormones^1^,^2^. The prevalence of subclinical thyroid disorders is between 10 and 15%, depending on previous cohorts. As subclinical hyperthyroid and hypothyroid disorders have unspecific symptoms, both are diagnosed by thyroxin levels normal range with abnormal TSH levels (decreased or slightly increased, respectively)^3^. Ethnicity, geographical factors, and iodine intake were reported to affect thyroid functions^4^.

Metabolic syndrome (MetS), which is also referred to as Syndrome X or insulin resistance syndrome, consists of several metabolic risk factors, including abdominal obesity, hypertension, dyslipidemia, and hyperglycemia. Its prevalence is increasing all over the world^5^.

While both MetS and hypothyroidism are independent risk factors for cardiovascular diseases, a growing body of evidence suggests that MetS components are affected by thyroid hormone imbalance^6^–^8^.

The aim of this study was to determine the prevalence of subclinical thyroid dysfunction in a group of apparently healthy Syrians and to evaluate the possible association between subclinical hypothyroidism and MetS.

## Methods

This retrospective cross-sectional study was conducted at Al-Assad University Hospital, Damascus, Syria (AUH) one of the considerable referral hospital in Syria. Secondary de-identified data was used in this study; it was part of a cross-sectional surveys conducted in AUH, Damascus, Syria, between 2010 and 2015 on apparently healthy participants^9^,^10^. The study protocol was approved by the Damascus University Review Board (DURB) and was in line with the STROCSS guideline^11^ (name of the registry: OSF preregistration, unique identifying number or registration ID: osf.io/tgxzv, and the hyperlink to this specific registration: https://archive.org/details/osf-registrations-tgxzv-v1).

A written informed consent was obtained from each participant and underwent a full clinical assessment.

Participants were recruited using a flyer advertising each study at the Department of Internal Medicine at AUH. Participants were from AUH staff or their relatives/friends or undergraduate/postgraduate students at Damascus Medical School. Pregnant and lactating women were excluded initially; 1577 volunteers were participated. Laboratory measurements were used to exclude participants who had impaired renal or liver function or intestinal malabsorption.

**Figure 1:**
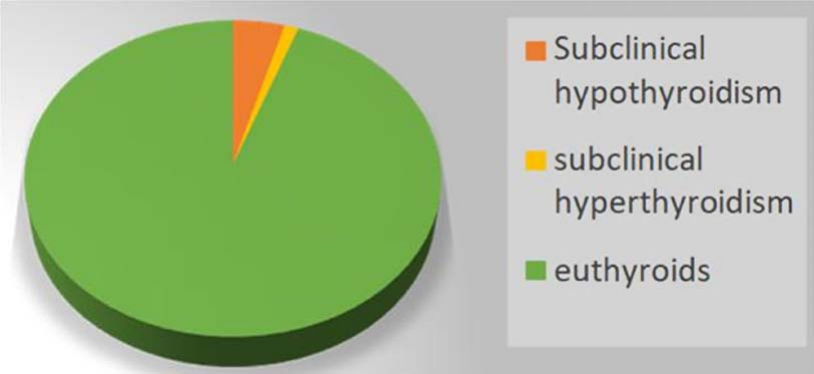
Flow chart showing participants selection and dropout.

Exclusion criteria also included previously diagnosed thyroid disease patients, patients who had thyroid surgery, and patients who were taking drugs affecting thyroid function tests (amiodarone, interferon, steroids, lithium, dopamine, levodopa, heparin, sertraline, high doses of aspirin ≥2 g/day, drugs containing iodine, and radiocontrast agents containing iodine within 4 weeks before the tests). The final sample comprised 1547 participants for the MetS study. However, participants who were taking hypoglycemic, dyslipidemic, and antihypertensive agents were also excluded, leaving 1111 participants (Fig. 1).

All biochemical tests were performed in the laboratory of the AUH. A venous blood sample was drawn from each participant after a 12-hour fast. Blood glucose, creatinine, liver enzymes, triglycerides (TGs), and high-density lipoprotein (HDL) were measured using an enzymatic colorimetric assay (Hitachi 912 device). Automated electrochemiluminescence immunoassay (Elecsys 2010 analyzers; Roche Diagnostics GmbH) was used for TSH (third generation) and FT4 measurement. While, antithyroid peroxidase (TPO) was measured using a quantitative enzyme-linked immunosorbent assay. Normal TSH range was 0.39–4.6 mU/l, FT4 range was 0.8–1.7 ng/dl, and anti-TPO range was 0–34 U/ml. Homeostatic Model Assessment for Insulin Resistance (HOMA-IR) was calculated according to the formula: fasting insulin (mU/l)×fasting glucose (nmol/l)/22.5. Values of HOMA-IR<2.60 were considered to be in the normal range, HOMA-IR 2.60–3.80 as ‘borderline high’, and HOMA-IR>3.80 as ‘high’ having clear correlates with insulin resistance^12^. Weight (with light clothes) and barefoot height were measured using the Seca Scale Model 713 device. BMI was calculated by the equation [BMI=weight (kg)/height (m^2^)]. Waist circumference (WC) was measured in centimeters using a soft scale at midway between the inferior costal margin and the iliac crest, horizontally parallel to the floor at the end of exhalation. Blood pressure (BP) was measured using the ALPK2 BP gauge.

Participants were categorized according to WHO criteria as follows: BMI less than 25 kg/m^2^ for normal weight, BMI greater than or equal to 25, below 30 kg/m^2^ for overweight, and BMI greater than or equal to 30 kg/m^2^ for obesity.

Subclinical hypothyroidism was diagnosed when peripheral thyroid hormone levels were within the normal laboratory reference range but TSH levels were mildly elevated (4.7–10 mU/l). Subclinical hyperthyroidism was diagnosed when peripheral thyroid hormone levels were within the normal laboratory reference range and TSH levels were mildly suppressed (0.4–0.1 mU/l). MetS was diagnosed based on the criteria of the International Diabetes Federation: central obesity (WC greater than or equal to 94 cm for men or greater than or equal to 80 cm for women) with two of the following^13^:

1. TG greater than or equal to 150 mg/dl.
2. HDL less than 40 mg/dl in males and less than 50 mg/dl in females.
3. Arterial pressure: systolic greater than or equal to 130 mm Hg, or diastolic greater than or equal to 85 mm Hg.
4. Fasting blood sugar greater than or equal to 100 mg/dl.

Data were analyzed using the SPSS software version 23.0 (IBM), *P* values less than 0.05 were considered statistically significant. Mean and SD was calculated. The *χ* ^2^-test was used for nominal data.

## Results

A total of 1111 participants were included in the study. Four hundred and thirty-four (39.1%) were males, and 677 (60.9%) were females. The mean age was 35.6±11.7 (years), mean BMI was 26.9±5.5 kg/m^2^, mean TSH was 2.13±1.38 mIU/ml, mean HDL was 50.68±13.92 mg/dl, mean TG was 119.84±81.26 mg/dl, mean diastolic BP was 72.7±10.3 mm Hg and the mean systolic BP was 116.6±14.2 mm Hg (Table 1).

**Table 1:** Description of numeric variables of the participants of the study.

Variables	*N*	Minimum	Maximum	Mean	SD
Age (years)	1111	18	82	35.68	11.773
TSH (mIU/ml)	1111	0.15	13.88	2.1362	1.38531
FT4 (ng/dl)	482	0.80	1.77	1.2215	0.16079
Anti-TPO (U/ml)	232	4.00	575.50	30.0102	75.78831
Waist (cm)	1111	51.50	130.00	87.8740	13.90961
Weight (kg)	891	1.65	144.00	73.8247	17.65706
Length (m)	889	1.40	190.00	4.3679	21.07246
BMI (kg/m^2^)	1102	14.69	59.94	26.9675	5.51651
Glucose (mg/dl)	1110	46	124	84.08	13.268
Insulin (mU/l)	874	0.20	51.55	8.8772	6.26063
HOMA-IR	873	0.024	15.020	1.85238	1.465983
HDL (mg/dl)	1101	17	138	50.68	13.911
TG (mg/dl)	1108	20	970	119.84	81.267
DBP (mm Hg)	1107	40	120	72.74	10.374
SBP (mm Hg)	1107	80	180	116.60	14.231

DBP, diastolic blood pressure; FT4, free thyroxin; HDL, high-density lipoprotein; HOMA-IR, Homeostatic Model Assessment for Insulin Resistance; SBP, systolic blood pressure; TG, triglycerides; TPO, thyroid peroxidase; TSH, thyroid-stimulating hormone.

**Figure 2:**
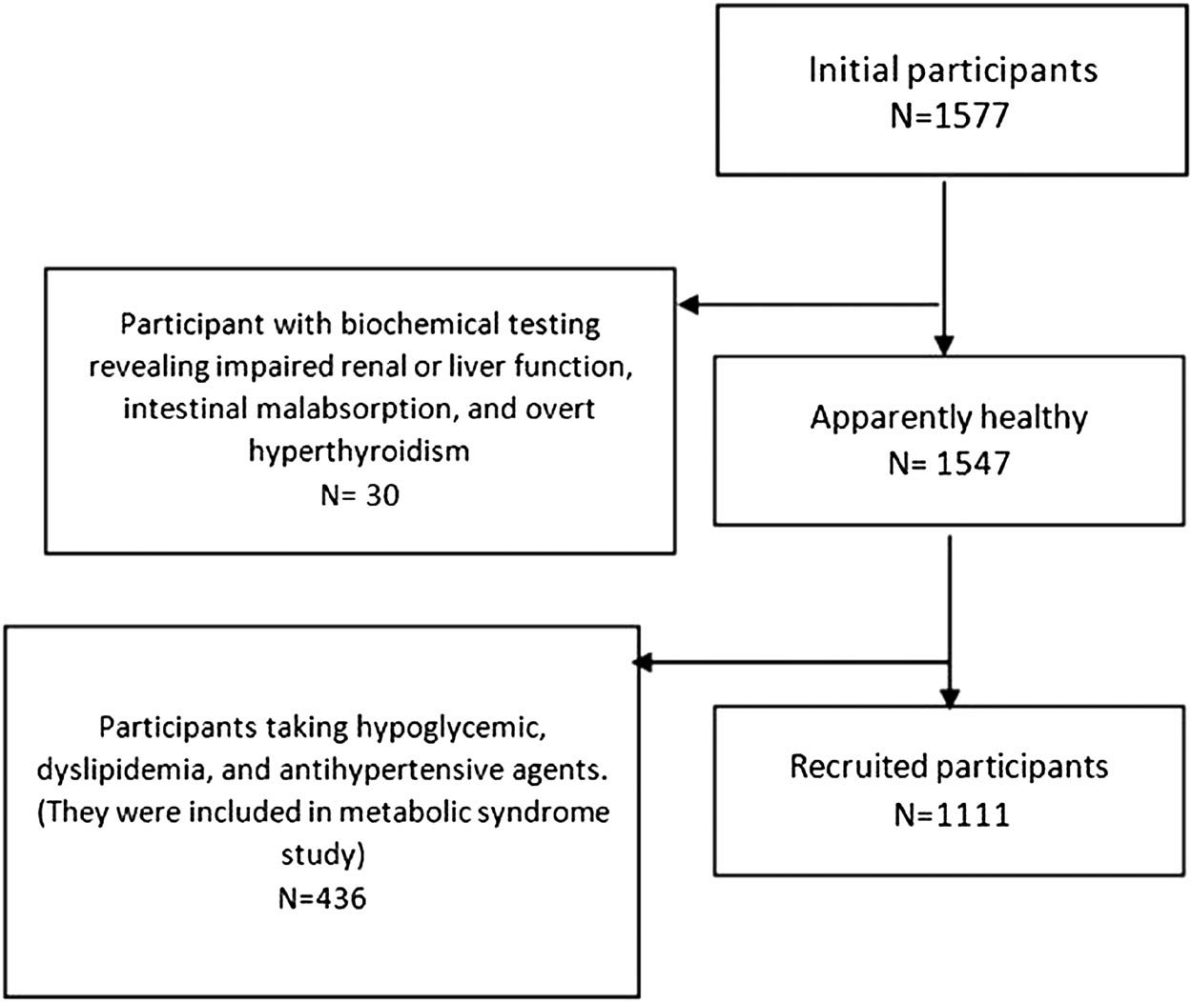
Distribution of participant according to thyroid function tests.

Subclinical hypothyroidism was present in 49 (4.4%) participants, and subclinical hyperthyroidism was present in 13 (1.2%) participants. While 1049 (94.4%) participants were in the euthyroid group (Fig. 2).

The overall prevalence of thyroid dysfunction was higher in females compared to males; where 84.6% of subclinical hyperthyroid and 65.3% of subclinical hypothyroid patients were females. The relationship between gender and thyroid dysfunction was significantly important (*P*=0.0001) (Table 2).

**Table 2:** Distribution of subclinical hypothyroidism, euthyroidism, and subclinical hyperthyroidism participants between male and female groups.

	Sex, *n* (%)		
	Male	Female	Total, *n* (%)
Thyroid status			
Subclinical hyperthyroidisms	2 (15.3)	11 (84.6)	13 (100)
Euthyroidism	415 (39.5)	634 (60.5)	1049 (100)
Subclinical hypothyroidism	17 (34.6)	32 (65.3)	49 (100)
Total	434 (39.1)	677 (60.9)	1111 (100)

Out of 232 participants who were tested for antithyroid peroxidase, 27 (11.5%) had high values that were considered to be positive. Twenty-one (77.7%) of them were euthyroid, and six (22.2%) of them had subclinical hypothyroidism (Table 3). The relative risk for having subclinical hypothyroidism when anti-TPO is positive was 11.5.

**Table 3:** Distribution of subclinical hypothyroidism, euthyroidism, and subclinical hyperthyroidism participants between positive and negative anti-TPO groups.

	Anti-TPO, *n* (%)	
	Negative	Positive
Thyroid status		
Subclinical hyperthyroidisms	2 (0.9)	0
Euthyroidism	199 (97)	21 (77.7)
Subclinical hypothyroidisms	4 (1.9)	6 (22.2)
Total	205 (100)	27 (100)

TPO, thyroid peroxidase.

Most of the subclinical hypothyroid participants (31 participants) had normal values of HOMA-IR, and only two participants had borderline high values, while the other two had values indicating the presence of insulin resistance. Due to the small number of participants who had abnormal values, no statistical tests were conducted. Most of the subclinical hypothyroid participants had normal values of fasting blood glucose, and only two had abnormal values (Table 4).

**Table 4:** Distribution of obesity, insulin resistance, and metabolic syndrome among euthyroid and subclinical hypothyroid participants.

	*N* (%)			
	Euthyroidism	Subclinical hypothyroidism	Total (*N*)	*P*
Weight groups				
Under weight	20 (1.9)	1 (2.04)	21	0.199
Normal	406 (38.8)	19 (38.7)	431	
Over weight	343 (32.8)	16 (32.6)	361	
Obesity	275 (26.3)	13 (26.5)	292	
Total	1044 (100)	49 (100)	1105	
HOMA-IR groups				
Normal	661 (79.6)	31 (88.5)	700	*
Border line high	102 (12.2)	2 (5.7)	104	
Insulin resistance	67 (8.07%)	2 (5.7)	70	
Total	830 (100)	35 (100)	874	
WC groups				
Normal	422 (**40.2**)	18 (36.7)	440	0.001
Central obesity	336 (32.1)	17 (**34.7**)	353	
Metabolic syndrome (≥2 risk factors)	291 (27.7)	14 (**28.6**)	305	
Total	1049 (100)	49 (100)	1098	

Bold value significance 0.001.

HOMA-IR, Homeostatic Model Assessment for Insulin Resistance; WC, waist circumference.

* No statistical test was conducted.

There was an increased prevalence of Mets among subclinical hypothyroid participants (28.5 versus 27.7%) among euthyroid participants (*P*=0.001) (Table 4).

Overweight and obesity (32.6 and 26.5%, respectively) in the subclinical hypothyroid group were comparable to those in the euthyroid group (32.8 and 26.3%, respectively) (Table 4). Although the prevalence of obesity was almost equal in both groups, a statistically significant difference (*P*=0.001) in central obesity was observed between subclinical hypothyroid and euthyroid patients (34.6 and 32%, respectively).

Table 5 shows the distribution of components of MetS among euthyroid and subclinical hypothyroid participants.

**Table 5:** Distribution of components of metabolic syndrome among euthyroid and subclinical hypothyroid participants.

	Euthyroidism, *N* (%)	Subclinical hypothyroidism, *N* (%)	*P*
DBP			
Abnormal	125 (11.8)	4 (8.1)	*
Normal	926 (88.1)	45 (91.8)	
Mean	72.71	73.23	
SBP			
Abnormal	244 (23.2)	7 (14.2)	*
Normal	807 (76.7)	42 (85.7)	
Mean	116.61	116.15	
Glucose			
Abnormal	117 (11.1)	2 (4)	*
Normal	934 (88.8)	47 (95.9)	
Mean	84.12	81.88	
TG			
Abnormal	259 (24.6)	15 (30.6)	0.007
Normal	791 (75.3)	34 (39.3)	
Mean	119.92	130.57	
HDL			
Abnormal	379 (36.3)	21 (42.8)	0.317
Normal	663 (63.6)	28 (57.1)	
Mean	50.64	50.16	
WC			
Abnormal	629 (59.7)	31 (63.2)	0.032
Normal	423 (40.2)	18 (36.7)	
Mean	87.83	87.98	

DBP, diastolic blood pressure ≥85 mm Hg; HDL, high-density lipoprotein, <40 mg/dl in males and <50 mg/dl in females; SBP, systolic blood pressure ≥130 mm Hg; TG, triglycerides ≥150 mg/dl; WC, waist circumference ≥94 cm (male) or ≥80 cm (female).

*No statistical test was conducted.

The mean diastolic BP of the subclinical hypothyroid participants was slightly higher compared with the euthyroid participants, however, the systolic BP was higher in the euthyroid group (Table 5).

However, there was a significant difference in impaired TG between the two groups, as 30.6% of the subclinical hypothyroid participant had TG greater than 150 mg/dl (mean: 130.57) whereas it was only 24.6% in the euthyroid group (mean: 119.92) (*P*=0.007).

Low HDL, a component of MetS when less than 40 mg/dl in males and less than 50 mg/dl in females, was more common in subclinical hypothyroid compared to euthyroid (42.8 and 36.3%, respectively); however, no significant difference was reported (*P*=0.317) and the mean HDL levels were nearly equal in both groups.

Finally, a significant association between increased WC and subclinical hypothyroidism was found (*P*=0.032), as 31 (63.2%) of the subclinical hypothyroid participants had WC greater than or equal to 94 cm (male) or greater than or equal to 80 cm (female) versus 629 (59.7%) of the euthyroid participants.

## Discussion

In this cross-sectional study of apparently healthy Syrians, a significant relationship between gender and thyroid dysfunction was noticed, where the overall prevalence of thyroid dysfunction (subclinical hypothyroidism and hyperthyroidism) was higher in females compared to males. There was a significant increase in MetS, TG levels, WC, and central obesity among the subclinical hypothyroid versus euthyroid group.

To the best of our knowledge, this is the first available data about the prevalence of these disorders in Syria, where the prevalence of subclinical hypothyroidism and hyperthyroidism was 4.4 and 1.2%, respectively. These results were comparable to global reports of subclinical hypothyroidism, which are in line with the range of ~4–10% of the general population and with a meta-analysis performed in Europe (4.11%)^14^,^15^. Yet were lesser than those reported by other studies performed in the USA (14.5%), the Netherlands (6%), and Korea (11.7–17.3% using data from two cohorts)^16^–^18^. On the other hand, the prevalence of subclinical hyperthyroidism resembles global studies that report 1–2% of the general population^17^,^19^.

Inconsistent data on the relation between subclinical hypothyroidism and MetS were published^6^–^8^,^20^–^22^. However, this relationship remains controversial. Many studies have reported the association of subclinical hypothyroidism with obesity, worsened lipid profiles, higher BP, and markers of systemic inflammation, and likewise MetS^21^,^22^. These studies mostly have a cross-sectional design and mostly do not having assessed MetS as a whole entity^7^. A striking result of a large cohort study of 66 822 Taiwanese participants reported a significant increased risk of developing subclinical hypothyroidism in MetS patients compared with non-MetS, and after controlling for risk factors, patients with MetS were at a 21% excess risk of developing subclinical hypothyroidism (adjusted hazard rate: 1.21)^6^.

In our study, MetS was found to be higher in subclinical hypothyroidism (28.5%) compared to euthyroid (27.7%) (*P*=0.001). Then by investigating the relationship between subclinical hypothyroidism and each component of MetS. However, the obesity was almost equal between the two groups, it was noticeable that the WC was higher in subclinical hypothyroidism (63.2% vs. 59.7%), in addition to a significant association with the presence of central obesity (34.6% vs. 32%) compared to the euthyroid group.

Several reports discussed the mechanism that links between obesity and hypothyroidism. High leptin levels, which are secreted by adipocytes, might play a role in stimulating thyrotropin and increasing thyroid susceptibility to autoimmunity with subsequent hypothyroidism^23^. On the other hand, adipose tissue secretes inflammatory cytokines such as tumor necrosis factor-α and interleukin (IL-1, IL-6) that impede the expression of sodium iodide transporter mRNA and the activity of iodine uptake in human thyroid cells, which reduce the secretion of thyroid hormones^24^.

Dyslipidemia is frequently observed in subclinical hypothyroidism^25^–^28^. Thyroid hormone has an important role in stimulating lipolysis in fat stores in white adipose tissue by activating lipoprotein lipase. Decreased thyroid hormone levels reduce sensitivity to catecholamine-induced lipolysis in brown adipose tissue^29^. As a result of decreased thyroid hormone, an accumulation of higher levels of TGs in the circulation could occur^30^. Furthermore, both IL-6 and tumor necrosis factor-α can promote lipodieresis^25^. Meanwhile, decreased thyroid hormone resulted in an increased level of HDL as a result of decreased activity of cholesteryl ester transfer protein and deactivating hepatic lipase, which hydrolyzes HDL2 to HDL3^31^. The data were mostly compatible in the relation of subclinical hypothyroidism with high levels of TGs^8^,^20^,^27^,^28^,^32^. By contrast, the relation of subclinical hypothyroidism was more conflict with HDL, somewhat revealed a low level of HDL^8^,^20^,^22^,^26^,^28^, and others showed high levels^31^,^32^. In our study, however, subclinical hypothyroidism did not correlate with HDL, and the mean level of TG was significantly higher compared to euthyroid (130.5 vs. 119.9).

The prevalence of subclinical hypothyroidism was lower in men than in women^7^,^15^. According to our study, the prevalence of thyroid dysfunction was higher in females compared to males, since 84.6% of subclinical hyperthyroid patients and 65.3% of subclinical hypothyroid patients were female. Sex disparity may be related to the different effects of sex hormones on thyroid metabolism, for example, the effects of estrogen on TSH and lipid profiles in women, in addition to the effect of low testosterone levels on MetS parameters in men^33^,^34^. Also, the risk of having to have autoimmune thyroid disease in women is about 10 times higher than in men. One reason for that is that thyroid disorders are often triggered by autoimmune responses, and women are more susceptible to autoimmune diseases than men^35^. This belief is partly attributed to the X chromosome, which contains many genes related to the immune system^36^.

An offsetting strength was that we were very strict in recruiting participants; inclusion criteria and examinations helped ensure that they were healthy and that the results were valid. While not representative, our sample is heterogeneous, as the university hospital is a big public referral hospital that Syrians from different areas seek for health care.

However, the limitation of this study was the small number of participants, which restricted the ability to apply appropriate statistical tests. As a result, the association between elevated systolic BP, elevated diastolic BP and impaired fasting blood glucose could not be evaluated.

## Conclusion

Among this group of apparently healthy Syrians, subclinical hypothyroidism was associated with increased MetS, TG levels, WC, and central obesity. Also, females were more susceptible to subclinical thyroid dysfunctions than males. Since MetS is a known factor for morbidity and mortality, this may raise the attention needed to perform future prospective trials to evaluate the possible benefits of subclinical hypothyroidism treatment with a low dose of thyroxin.

## Ethical approval

The study was approved by the Damascus University Review Board (DURB), in accordance with the Declaration of Helsinki, and in line with the STROCSS criteria.

## Consent

Secondary de-identified data was used in this study; it was part of cross-sectional surveys conducted in AUH, Damascus, Syria, between 2010 and 2015 on apparently healthy participants (written informed consent was obtained from each participant in those surveys).

## Sources of funding

This research did not receive any specific grant from funding agencies in the public, commercial, or not-for-profit sectors.

## Author contributions

Z.A.: supervised data collection, conceptualized and design the study, wrote the study protocol, did the literature search, and drafted the manuscript. N.H.: participated in the statistical analysis, the design, and writing the first draft. M.A.: participated in writing the final copy and final submission. All authors read and approved the final copy.

## Conflicts of interest disclosure

The author declares that they have no conflicts of interest regarding this study. The author declares that it has not been published elsewhere and that it has not been simultaneously submitted for publication elsewhere.

## Research registration unique identifying number (UIN)

1. Name of the registry: OSF Preregistration.
2. Unique identifying number or registration ID: osf.io/tgxzv.
3. Hyperlink to your specific registration (must be publicly accessible and will be checked): https://archive.org/details/osf-registrations-tgxzv-v1

## Guarantor

Zaynab Alourfi.

## Provenance and peer review

Not commissioned, externally peer-reviewed.
